# Relationship between phthalates exposures and metabolic dysfunction-associated fatty liver disease in United States adults

**DOI:** 10.1371/journal.pone.0301097

**Published:** 2024-04-19

**Authors:** Junhao Sun, Siqi Yang, Yue Zhang, Wenzhi Xiang, Xiubo Jiang

**Affiliations:** 1 Department of Epidemiology and Health Statistics, The School of Public Health of Qingdao University, Qingdao, China; 2 Qingdao Maternal & Child Health and Family Planning Service Center, Qingdao, China; 3 Huangdao District Center for Disease Control and Prevention, Qingdao, China; Shahjalal University of Science and Technology, BANGLADESH

## Abstract

As a new definition for the evidence of hepatic steatosis and metabolic dysfunctions, the relationship between phthalates (PAEs) and metabolic dysfunction-associated fatty liver disease (MAFLD) remains virtually unexplored. This study included 3,137 adults from the National Health and Nutrition Examination Survey spanning 2007–2018. The diagnosis of MAFLD depended on the US Fatty Liver Index (US FLI) and evidence of metabolic dysregulation. Eleven metabolites of PAEs were included in the study. Poisson regression, restricted cubic spline (RCS), and weighted quantile sum (WQS) regression were used to assess the associations between phthalate metabolites and MAFLD. After adjusting for potential confounders, Poisson regression analysis showed that mono-2-ethyl-5-carboxypentyl phthalate (MECPP), mono-n-butyl phthalate, mono-(3-carboxypropyl) phthalate, mono-ethyl phthalate (MEP), mono-(2-ethyl-5-hydroxyhexyl) phthalate (MEHHP) and mono-(2-ethyl-5-oxohexyl) phthalate were generally significant positively associated with MAFLD (*P*<0.05). Furthermore, the WQS index constructed for the eleven phthalates was significantly related to MAFLD (OR:1.43; 95%CI: 1.20, 1.70), MEHHP (33.30%), MEP (20.84%), MECPP (15.43%), and mono-isobutyl phthalate (11.78%) contributing the most. This study suggests that exposure to phthalates, individually or in combination, may be associated with an increased risk of MAFLD.

## Introduction

Metabolic dysfunction-associated fatty liver disease (MAFLD) was proposed by a panel of international experts from 22 countries as a new definition to replace non-alcoholic fatty liver disease (NAFLD) in 2020. MAFLD reflects the hepatic manifestation of a multisystem disorder, although its underlying causes, presentation, duration, and outcome are different from NAFLD [1]. Unlike NAFLD, which calls for ruling out other chronic liver diseases and alcohol consumption, the diagnosis of MAFLD is made using positive diagnostic criteria. These criteria include the presence of hepatic steatosis in addition to one of three factors: overweight/obesity, type 2 diabetes mellitus (T2DM), or metabolic dysregulation [1]. While the abbreviation NAFLD is still in use, more recent studies are adopting MAFLD, which employs a positive definition [2]. A recent meta-analysis found that the prevalence of MAFLD is 39.0% in the general population [3]. Though the disease causes a substantial health and economic burden on many countries, there are no approved drug treatments yet [3,4].

Phthalates (PAEs), employed extensively as plasticizers (comprising approximately 80% to 85% of all plasticizers), are incorporated into plastics to enhance flexibility, processability, and extensibility [5,6]. Apart from being employed as plasticizers, PAEs are also utilized in various personal care products, including perfumes, soaps, and other items for fragrance purposes [7]. Due to the absence of chemical bonds to the polymer matrix, phthalate plasticizers are prone to leaching or migrating from the plastic into the environment, influenced by factors like pH, temperature, pressure, irradiation, and exposure to lipids or solvents [8]. These compounds are pervasive across different settings, facilitating their migration and accumulation within the food chain, thereby exposing humans through dietary consumption, dermal absorption, and the inhalation of air, both indoors and outdoors [9]. Globally recognized for their estrogen-mimicking properties and mutagenic, teratogenic, and carcinogenic effects on human health, PAEs are classified as endocrine-disrupting chemicals (EDCs) [10]. PAEs imitate the function of natural endogenous estrogens, and when they bind to human estrogen receptors (ERs), they disrupt hormone signaling pathways [11]. Based on previous research, as a class of EDCs, PAEs are associated with reproductive system malfunctions and liver toxicity, among other adverse effects [12].

In several animal studies, researchers have observed that exposure to PAEs leads to increased hepatic inflammation in adult zebrafish and elevated blood glucose and insulin levels in mice [13–16]. Additionally, an animal study has demonstrated that diethyl phthalate (DEP), a type of PAE, induces significant lipid peroxidation in the livers of treated rats [17]. In 2021, a cross-sectional study revealed a positive correlation between urinary concentrations of phthalate metabolites and alterations in liver function test markers, including alanine aminotransferase (ALT), aspartate aminotransferase (AST), gamma-glutamyl transferase (GGT), and alkaline phosphatase (ALP) [18]. Elevated ALT and AST activities serve as markers for liver dysfunction in clinical practice [19]. Moreover, GGT serves as a reliable predictor of liver mortality and is utilized in clinical settings to identify the hepatic origin of increased ALP levels, which are crucial indicators of liver dysfunction [20,21]. A study reported that the combined effect of phthalates on glucose and lipid metabolism may enhance the risk of NAFLD and insulin resistance development in exposed individuals [22].

To our knowledge, there have been limited epidemiological studies that specifically investigate the association between phthalates and MAFLD. Certain research indicates a potential link between PAEs, particularly di-2-ethylhexyl phthalate (DEHP), and NAFLD [23]. Unlike NAFLD, the diagnosis of MAFLD takes into account metabolic dysfunction, obesity, and T2DM. Previous studies have found clear differences in the natural progression, prevalence, baseline characteristics, and severity scores between NAFLD and MAFLD patients [24]. Phthalates have been reported as risk factors for obesity development in the general population [25]. Furthermore, exposure to phthalates has been linked to an elevated risk of developing metabolic syndrome [26,27]. Hence, it is necessary to explore the potential connection between phthalate exposure and MAFLD. In this study, we aimed to investigate the relationship between urinary phthalate metabolites and MAFLD in American adults.

## Methods

### Study population

The survey and consent documents for the National Health and Nutrition Examination Survey (NHANES) received approval from the Institutional Review Board at the Centers for Disease Control and Prevention (CDC). The National Center for Health Statistics’ (NCHS) Research Ethics Review Committee also approved the study protocol (approval number: #2005–06, #2011–17, #2018–01), and the participants gave their written informed permission. Initiated in the early 1960s, NHANES is a repeated cross-sectional survey program that employs multistage sampling strategies to assess the dietary and health status of Americans. Conducted biennially, the survey utilizes a sophisticated sampling design to select specific population subgroups, applying survey weights to ensure the sample is nationally representative. NHANES encompasses interviews, health examinations, and laboratory tests. Participants initially underwent home interviews to collect background information, encompassing socio-demographic, medical, and family histories. Subsequently, they visited the Mobile Examination Center (MEC) to provide additional relevant data, including anthropometric measurements and laboratory assessments.

**Fig 1:**
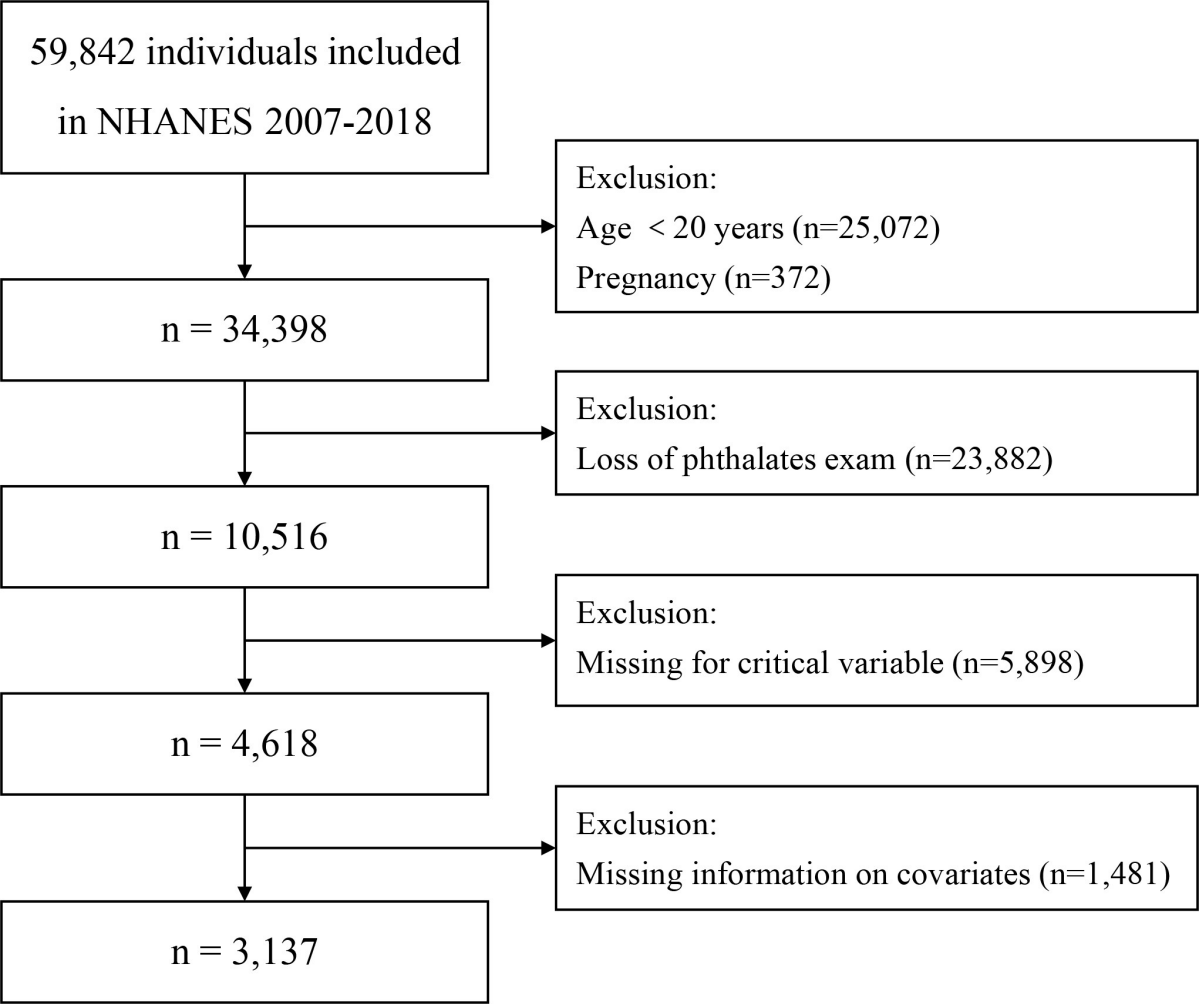
Flow chart for study population selection from NHANES 2007–2018.

Data from six continuous cycles of the NHANES were used (2007–2008, 2009–2010, 2011–2012, 2013–2014, 2015–2016, and 2017–2018). The exclusion criteria comprised: (1) individuals under 20 years of age; (2) females who were pregnant; (3) individuals lacking essential data for crucial variables; (4) individuals with missing information on covariates. Ultimately, 3,137 individuals were considered eligible for analysis (Fig 1).

### Phthalates examination

Spot urine samples were obtained at MECs. Within the MEC, the process of biospecimen collection included the retrieval, processing, storage, and shipping of various samples, ensuring standardized testing. The samples were stored at -20°C until analysis. Phthalate metabolites in urine samples were extracted using solid-phase extraction and quantified via high-performance liquid chromatography-electrospray ionization-tandem mass spectrometry (HPLC-ESI-MS/MS). For detailed methodologies, please refer to the Laboratory Procedure Manual available on the NHANES website.

In this study, We analyzed 11 urinary phthalate metabolites: mono(carboxynonyl) phthalate (MCiNP), mono(carboxyoctyl) phthalate (MCiOP), mono-2-ethyl-5-carboxypentyl phthalate (MECPP), mono-n-butyl phthalate (MnBP), mono-(3-carboxypropyl) phthalate (MCPP), mono-ethyl phthalate (MEP), mono-(2-ethyl-5-hydroxyhexyl) phthalate (MEHHP), mono-(2-ethylhexyl) phthalate (MEHP), mono-isobutyl phthalate (MiBP), mono-(2-ethyl-5-oxohexyl) phthalate (MEOHP), mono-benzyl phthalate (MBzP). For concentrations below the limit of detection (LOD), NCHS recommends dividing them by the square root of two.

### Definition of MAFLD

The proposed MAFLD criteria rely on histological (biopsy), imaging, or blood biomarker evidence indicating hepatic steatosis, coupled with one of the following: overweight/obesity, T2DM, or signs of metabolic dysregulation [1]. We used the United States Fatty Liver Index (US FLI) as a predictor of hepatic steatosis. The US FLI was calculated using NHANES data, achieving an area under the receiver operating characteristic curve (AUROC) of 0.78, effectively predicting NAFLD as confirmed by ultrasound [28]. The US FLI is as follows:

$$\mathrm{US}\;\mathrm{FLI}=\frac{e^{‐0.8073\times \mathrm{non}‐\mathrm{Hispanic}\;\mathrm{black}+0.3458\times \mathrm{Mexican}\;\mathrm{American}+0.0093\times \mathrm{age}+0.6151\times \log_{e}(\mathrm{GGT})+0.0249\times \mathrm{waist}+1.1792\times \log_{e}(\mathrm{insulin})+0.8242\times \log_{e}(\mathrm{glucose})‐14.7812}}{\left(1+e^{‐0.8073\times \mathrm{non}‐\mathrm{Hispanic}\;\mathrm{black}+0.3458\times \mathrm{Mexican}\;\mathrm{American}+0.0093\times \mathrm{age}+0.6151\times \log_{e}(\mathrm{GGT})+0.0249\times \mathrm{waist}+1.1792\times \log_{e}(\mathrm{insulin})+0.8242\times \log_{e}(\mathrm{glucose})‐14.7812}\right)}\times 100$$

Participants with a US FLI score ≥ 30 were categorized as having liver steatosis.

Overweight/obesity was defined as a body mass index (BMI) of 25kg/m^2^ or higher. T2DM was diagnosed if fasting glucose levels were 126 mg/dl or higher, or hemoglobin A1c (HbA1c) was 6.5% or above, having either diagnosed diabetes by the doctor or glucose-lowering medications using by self-report questionnaire [29]. Metabolic dysregulation was identified by the presence of at least two of the following criteria: (1) Waist circumference (WC) of 102 cm or more for Caucasian men and 88 cm or more for Caucasian women (or 90 cm or more for Asian men and 80 cm or more for Asian women); (2) Blood pressure of 130/85 mmHg or higher or treatment with specific antihypertensive drugs; (3) Plasma triglycerides of 150 mg/dl (1.70 mmol/L) or higher or treatment with specific lipid-lowering drugs; (4) Plasma high density lipoprotein cholesterol (HDL-c) levels less than 40 mg/dl (1.0 mmol/L) for men and less than 50 mg/dl (1.3 mmol/L) for women or treatment with specific lipid-lowering drugs; (5) Prediabetes, defined as fasting glucose levels between 100 to 125 mg/dl (5.6 to 6.9 mmol/L), 2-hour post-load glucose levels between 140 to 199 mg/dl (7.8 to 11.0 mmol/L), or HbA1c between 5.7% to 6.4% (39 to 47 mmol/mol); (6) Homeostasis Model Assessment of Insulin Resistance (HOMA-IR) score of 2.5 or higher; (7) Plasma high-sensitivity C-reactive protein (hs-CRP) level greater than 2 mg/L [1].

### Covariates

Based on existing literature, we included the following potential confounding factors as covariates: age, sex, race/ethnicity, poverty income ratio (PIR), education levels, smoking status, alcohol consumption, physical activity, creatinine, and the cycle of NHANES. S1 Table shows the detailed classification of covariates.

### Statistical analysis

Data were analyzed using R version 4.3.0 (R Foundation for Statistical Computing, Vienna, Austria) and STATA 15.0 (Stata Corporation, College Station, TX, USA). A two-sided *P*-value of <0.05 was considered statistically significant. For the complex sampling design of NHANES, the proper weight was used in this study. Categorical variables were presented using numbers (percentages) and continuous variables as medians (interquartile ranges). The Chi-square test and Kruskal–Wallis test were used to test the difference between non-MAFLD and MAFLD groups.

Due to skewed distributions, PAE levels were divided into quartiles, with quartile 1 (Q1) as the reference, after being transformed by natural logarithm. We employed Poisson regression to examine the association between PAE levels and MAFLD, calculating relative risks (RRs) and 95% confidence intervals (95% CIs). We used the number of people included in each cycle of this study as an offset. Only age and sex were adjusted in Model 1. Model 2 was further adjusted for race/ ethnicity, educational level, smoking status, alcohol consumption, physical activity, PIR, creatinine, and cycle. Finally, restricted cubic spline (RCS) with three knots at the 5th, 50th, and 95th percentiles was used to investigate the dose-response association between PAE concentration and risk of MAFLD.

The “gWQS” package (3.0.4) was used to perform weighted quantile sum (WQS) regression to assess the effects of environmental pollutant mixtures. Each phthalate metabolite received a weight between 0 and 1, cumulatively equating to 1. The WQS index consisted of the weighted sums of individual phthalate concentrations. The weights represent the relative contribution of each element in the mixture or the proportions of the chemical components that create an association [30]. Whereas, the weight of phthalate metabolites could be valuable only if the association between the WQS index and MAFLD was statistically significant [31]. Prior to regression analysis, PAE metabolite concentrations underwent natural logarithm transformation to achieve a closer approximation to normal distribution. In the WQS regression model, PAE concentrations were first categorized into quartiles based on existing literature. Subsequently, a training set comprising 40% randomly sampled data was employed, while the remaining 60% served for model validation. The association between the WQS index of the mixture and MAFLD was derived in positive and negative directions and adjusted for the same covariates, using 1000 bootstrap samples.

## Results

### Population characteristics

Table 1 shows the characteristics of a total of 3,137 participants. Among them, 1056 (33.66%) participants were determined with MAFLD. Participants with MAFLD tended to be older and more likely to be male, had lower levels of education, were obese, and had HBP (High blood pressure) or T2DM compared to those without MAFLD. Significant differences were also observed in race/ethnicity, physical activity, and smoking status. S2 Table shows more information about the general characteristics of the participants.

**Table 1:** General characteristics of the participants.

Variables	Total	non-MAFLD	MAFLD	*P* value
	n = 3,137	n = 2,081	n = 1,056	
**Age (years), N(%)**				<0.001
20–39	1116(35.58)	846(40.65)	270(25.57)	
40–59	1083(34.52)	686(32.96)	397(37.59)	
≥60	938(29.90)	549(26.38)	389(36.84)	
**Sex, N(%)**				<0.001
Male	1587(50.59)	988(47.48)	599(56.72)	
Female	1550(49.41)	1093(52.52)	457(43.28)	
**Race/ ethnicity, N(%)**				<0.001
Mexican American	437(13.93)	180(8.65)	257(24.34)	
Other Hispanic	310(9.88)	194(9.32)	116(10.98)	
Non-Hispanic White	1370(43.67)	914(43.92)	456(43.18)	
Non-Hispanic Black	640(20.40)	506(24.32)	134(12.69)	
Other Race	380(12.11)	287(13.79)	93(8.81)	
**Education, N(%)**				0.007
<high school	650(20.72)	366(17.59)	284(26.89)	
high school or equivalent	692(22.06)	463(22.25)	229(21.69)	
>high school	1795(57.22)	1252(60.16)	543(51.42)	
**PIR, N(%)**				0.741
Low	938(29.90)	601(28.88)	337(31.91)	
Middle	1122(35.77)	743(35.70)	379(35.89)	
High	1077(34.33)	737(35.42)	340(32.20)	
**Smoking status, N(%)**				0.008
Non-smoker	1779(56.71)	1215(58.39)	564(53.41)	
Previous smoker	657(20.94)	382(18.36)	275(26.04)	
Current smoker	701(22.35)	484(23.26)	217(20.55)	
**Alcohol, N(%)**				0.109
never	482(15.36)	304(14.61)	178(16.86)	
moderate	1334(42.52)	906(43.54)	428(40.53)	
heavy	1321(42.11)	871(41.85)	450(42.61)	
**Physical activity, N(%)**				<0.001
sedentary	708(22.57)	421(20.23)	287(27.18)	
insufficient	366(11.67)	230(11.05)	136(12.88)	
Moderate	342(10.90)	210(10.09)	132(12.50)	
High	1721(54.86)	1220(58.63)	501(47.44)	
**HBP, N(%)**				<0.001
No	1649(52.57)	1260(60.55)	389(36.84)	
Yes	1488(47.43)	821(39.45)	667(63.16)	
**T2DM, N(%)**				<0.001
No	2513(80.11)	1868(89.76)	645(61.08)	
Yes	624(19.89)	213(10.24)	411(38.92)	
**Cycle, N(%)**				0.800
2007–2008	531(16.93)	356(17.11)	175(16.57)	
2009–2010	563(17.95)	360(17.30)	203(19.22)	
2011–2012	488(15.56)	322(15.47)	166(15.72)	
2013–2014	563(17.95)	384(18.45)	179(16.95)	
2015–2016	513(16.35)	343(16.48)	170(16.10)	
2017–2018	479(15.27)	316(15.19)	163(15.44)	
**BMI (kg/m**^**2**^**)** ^**a**^	27.82(8.60)	25.81(6.30)	33.10(8.77)	<0.001
**Creatinine (mg/dl)** ^**a**^	117.00(98.00)	115.00(102.00)	122.00(92.00)	<0.001

PIR, poverty income ratio; HBP, high blood pressure; T2DM, type 2 diabetes mellitus; BMI, body mass index.

^a^ Continuous variables, presented as median (interquartile range).

### Concentration distribution of PAEs

Table 2 presents the PAE concentration distribution in the different ages and sexes. The median and interquartile ranges (IQR) of concentrations (ng/ml) of MCiNP, MCiOP, MECPP, MnBP, MCPP, MEP, MEHHP, MEHP, MiBP, MEOHP, and MBzP among all participants were 2.10(3.10), 9.30(21.30), 13.00(19.00), 12.20(17.60), 1.90(3.10), 48.77(120.09), 7.90(12.80), 1.16(2.03), 8.20(11.60), 5.10(7.70), and 4.70(9.28), respectively. Statistical differences in the distribution of MCiNP, MCiOP, MECPP, MCPP, MEHHP, MEHP, and MEOHP were observed across different age and sex strata (*P*<0.05). MiBP and MBzP showed significant differences only across age strata (*P*<0.05).

**Table 2:** The concentration distribution [Median (IQR)] of urinary PAEs (ng/mL) in different ages, sexes.

Lamination	MCiNP	MCiOP	MECPP	MnBP	MCPP	MEP	MEHHP	MEHP	MiBP	MEOHP	MBzP
**All participants**	2.10(3.10)	9.30(21.30)	13.00(19.00)	12.20(17.60)	1.90(3.10)	48.77(120.09)	7.90(12.80)	1.16(2.03)	8.20(11.60)	5.10(7.70)	4.70(9.28)
**Age (years)**											
20–39	2.40(3.47)	10.95(28.55)	13.95(21.10)	13.55(18.15)	2.00(3.70)	49.90(111.82)	8.50(14.11)	1.50(2.63)	9.90(13.88)	5.52(8.75)	6.17(13.04)
40–59	2.10(3.00)	8.70(20.90)	12.40(17.60)	12.20(18.40)	1.90(3.20)	47.85(132.20)	7.90(12.57)	1.10(1.93)	8.20(11.20)	4.90(7.10)	4.70(8.32)
≥60	2.00(2.60)	8.15(16.95)	12.80(18.92)	11.34(15.60)	1.65(2.70)	48.92(121.51)	7.40(11.70)	0.80(1.35)	6.50(9.20)	4.90(7.60)	3.51(6.00)
	<0.001	<0.001	0.027	0.05	0.003	0.661	0.01	<0.001	<0.001	0.004	<0.001
**Sex**											
Male	2.35(3.30)	9.63(23.50)	13.80(20.33)	11.90(15.82)	2.07(3.50)	46.87(122.80)	8.90(13.80)	1.30(2.33)	8.40(10.70)	5.50(8.30)	4.80(8.77)
Female	1.90(2.80)	8.70(19.80)	12.10(18.00)	12.75(19.40)	1.70(2.93)	50.46(116.20)	7.03(11.70)	1.00(1.73)	7.90(12.50)	4.70(7.30)	4.60(9.62)
	<0.001	0.017	<0.001	0.222	<0.001	0.318	<0.001	<0.001	0.188	<0.001	0.393

IQR: Interquartile range; MCiNP: Mono(carboxynonyl) phthalate; MCiOP: Mono(carboxyoctyl) phthalate; MECPP: Mono-2-ethyl-5-carboxypentyl phthalate; MnBP: Mono-n-butyl phthalate; MCPP: Mono-(3-carboxypropyl) phthalate; MEP: Mono-ethyl phthalate; MEHHP: Mono-(2-ethyl-5-hydroxyhexyl) phthalate; MEHP: Mono-(2-ethyl)-hexyl phthalate; MiBP: Mono-isobutyl phthalate; MEOHP: Mono-(2-ethyl-5-oxohexyl) phthalate; MBzP: Mono-benzyl phthalate.

### The correlation of phthalate metabolites

**Fig 2:**
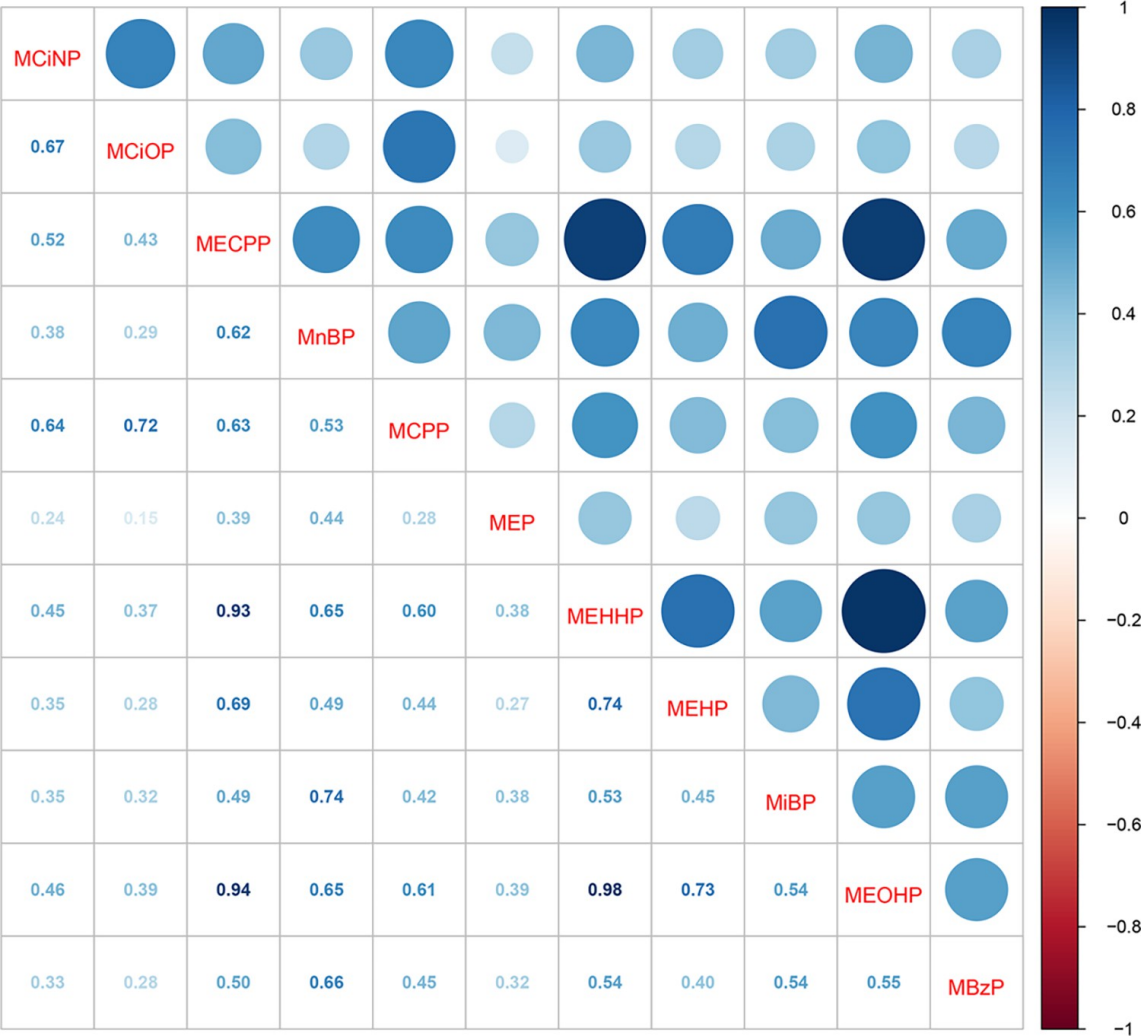
Pairwise correlation between eleven phthalate metabolites.

All phthalate metabolites were significantly and positively correlated with each other (Fig 2, all *P* values<0.05). Strong positive associations were observed between MECPP and MEHHP (r = 0.98, *P*<0.001), as well as between MECPP and MEOHP (r = 0.94, *P*<0.001).

### Association of PAEs with MAFLD

Eleven urinary phthalate metabolites were compared with MAFLD using Poisson regression, adjusting for relevant covariates (Table 3). In Model 2, the analysis was adjusted for age, sex, race, education level, PIR, smoking status, alcohol consumption, physical activity, creatinine, and cycle. There were significant associations between phthalate metabolites and MAFLD, with the exception of MEHP.

**Table 3:** Relationship between phthalates and MAFLD.

PAEs		model1^a^		model2^b^	
		RR (95% CI)	*P*-trend	RR (95% CI)	*P*-trend
**MCiNP**	Q1	Ref.	0.003	Ref.	0.186
	Q2	1.43(1.14,1.79)**		1.28(1.01,1.61)*	
	Q3	1.50(1.20,1.87)**		1.31(1.03,1.67)*	
	Q4	1.41(1.11,1.79)**		1.21(0.93,1.58)	
**MCiOP**	Q1	Ref.	0.003	Ref.	0.233
	Q2	1.51(1.25,1.82)**		1.33(1.08,1.64)**	
	Q3	1.27(0.99,1.63)		1.10(0.84,1.45)	
	Q4	1.50(1.21,1.85)**		1.29(1.00,1.67)	
**MECPP**	Q1	Ref.	<0.001	Ref.	0.012
	Q2	1.48(1.20,1.82)**		1.32(1.04,1.68)*	
	Q3	1.54(1.24,1.91)**		1.36(1.05,1.77)*	
	Q4	1.63(1.27,2.10)**		1.48(1.09,2.00)*	
**MnBP**	Q1	Ref.	<0.001	Ref.	0.03
	Q2	1.39(1.14,1.68)**		1.30(1.05,1.60)*	
	Q3	1.50(1.21,1.87)**		1.36(1.04,1.76)*	
	Q4	1.63(1.30,2.05)**		1.43(1.04,1.97)*	
**MCPP**	Q1	Ref.	<0.001	Ref.	0.085
	Q2	1.55(1.24,1.95)**		1.41(1.10,1.80)**	
	Q3	1.52(1.24,1.85)**		1.34(1.07,1.68)*	
	Q4	1.50(1.19,1.88)**		1.32(1.00,1.73)*	
**MEP**	Q1	Ref.	<0.001	Ref.	0.044
	Q2	1.44(1.18,1.77)**		1.34(1.09,1.66)**	
	Q3	1.42(1.13,1.78)**		1.29(1.02,1.65)*	
	Q4	1.50(1.21,1.87)**		1.35(1.07,1.71)*	
**MEHHP**	Q1	Ref.	<0.001	Ref.	<0.001
	Q2	1.44(1.22,1.70)**		1.31(1.08,1.59)**	
	Q3	1.58(1.30,1.93)**		1.43(1.14,1.79)**	
	Q4	1.56(1.25,1.95)**		1.49(1.15,1.92)**	
**MEHP**	Q1	Ref.	0.243	Ref.	0.501
	Q2	1.01(0.82,1.23)		0.97(0.79,1.20)	
	Q3	1.17(0.95,1.43)		0.98(0.80,1.20)	
	Q4	1.08(0.86,1.35)		0.92(0.74,1.14)	
**MiBP**	Q1	Ref.	<0.001	Ref.	0.048
	Q2	1.20(1.01,1.42)*		1.11(0.93,1.32)	
	Q3	1.65(1.34,2.02)**		1.45(1.16,1.81)**	
	Q4	1.45(1.16,1.82)**		1.23(0.92,1.64)	
**MEOHP**	Q1	Ref.	<0.001	Ref.	0.009
	Q2	1.68(1.39,2.03)**		1.51(1.21,1.88)**	
	Q3	1.54(1.29,1.84)**		1.36(1.10,1.68)**	
	Q4	1.64(1.30,2.06)**		1.48(1.13,1.94)**	
**MBzP**	Q1	Ref.	<0.001	Ref.	0.01
	Q2	1.32(1.08,1.61)**		1.24(1.01,1.54)*	
	Q3	1.26(1.03,1.54)*		1.17(0.92,1.49)	
	Q4	1.67(1.39,2.01)**		1.54(1.16,2.03)**	

Calculated using Poisson models.

^a^ Model 1 adjusted for age and sex.

^b^ Model 2 adjusted for age, sex, race/ethnicity, education level, PIR, smoke status, alcohol consumption, physical activity, creatinine, and cycle. *P<0.05

**P<0.01.

Compared with Q1, individuals in the higher quartiles of MECPP (RR,95%CI: Q4: 1.48, 1.09–2.00; Q3: 1.36, 1.05–1.77; Q2: 1.32, 1.04–1.68; *P*-trend = 0.012), MnBP (RR,95%CI: Q4: 1.43, 1.04–1.97; Q3: 1.36, 1.04–1.76; Q2: 1.30, 1.05–1.60; *P*-trend = 0.03), MCPP (RR,95%CI: Q4: 1.32, 1.00–1.73; Q3: 1.34, 1.07–1.68; Q2: 1.41, 1.10–1.80; *P*-trend = 0.085), MEP (RR,95%CI: Q4: 1.35, 1.07–1.71; Q3: 1.29, 1.02–1.65; Q2: 1.34, 1.09–1.66; *P*-trend = 0.044), MEHHP (RR,95%CI: Q4: 1.49, 1.15–1.92; Q3: 1.43, 1.14–1.79; Q2: 1.31, 1.08–1.59; *P*-trend<0.001), MEOHP (RR,95%CI: Q4: 1.48, 1.13–1.94; Q3: 1.36, 1.10–1.68; Q2: 1.51, 1.21–1.88; *P*-trend = 0.009) were significant positively associated with MAFLD.

### Dose-response relationships between PAEs and MAFLD

**Fig 3:**
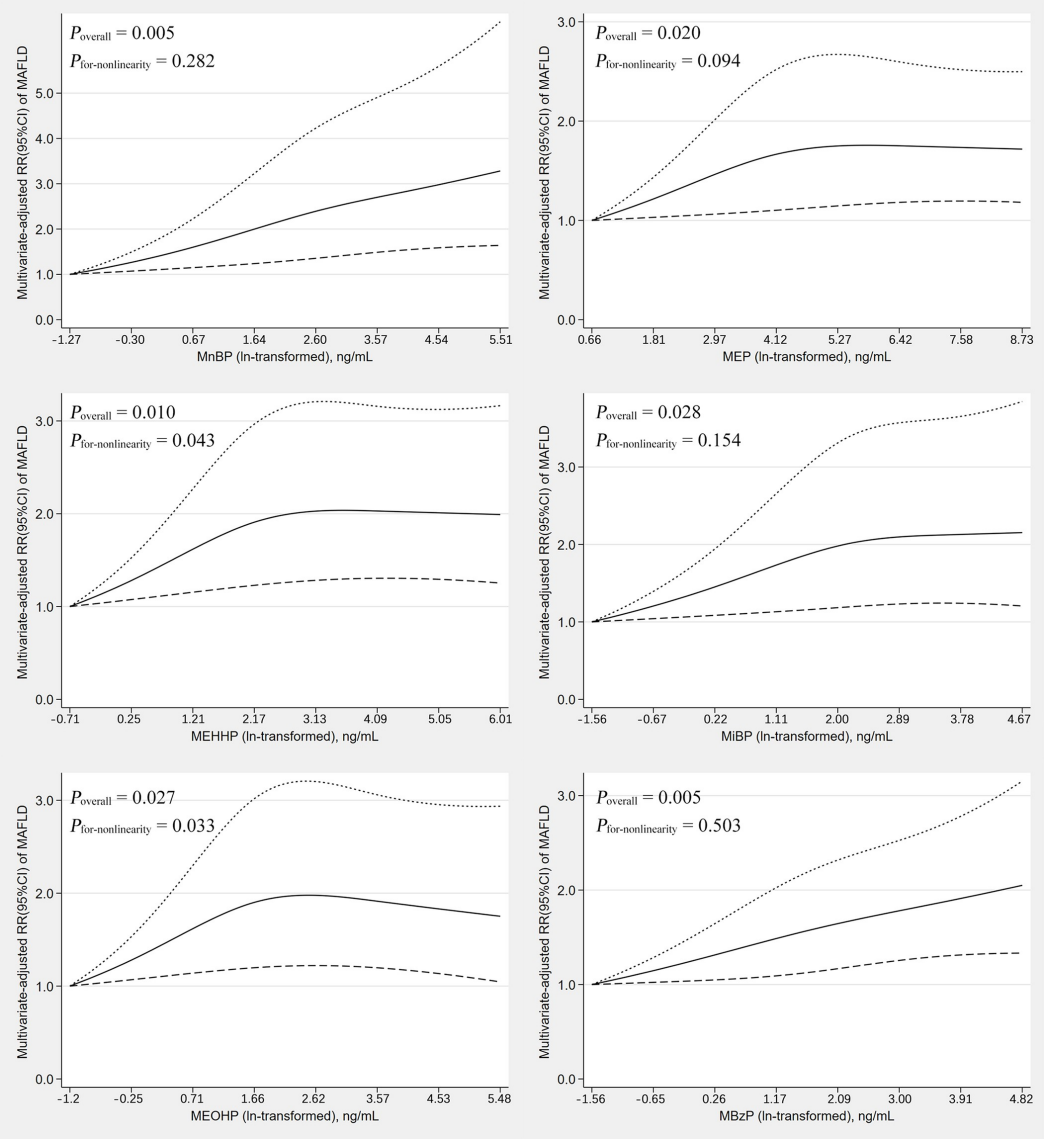
Associations between PAE concentration and MAFLD in Restricted Cubic Spline model for the overall population. The solid line and dashed lines represent the estimated RRs and the 95%CI.

The dose-response association between single phthalate metabolite and MAFLD was shown using the adjusted RCS models. We found that MnBP (*P*_overall_ = 0.005), MEP (*P*_overall_ = 0.020), MEHHP (*P*_overall_ = 0.010), MiBP (*P*_overall_ = 0.028), MEOHP (*P*_overall_ = 0.027) and MBzP (*P*_overall_ = 0.005) were significantly associated with MAFLD (Fig 3). Nonlinear dose-response relationships were observed between MEHHP (*P*_for-nonlinearity_ = 0.043), and MEOHP (*P*_for-nonlinearity_ = 0.033) with MAFLD. Restrictive cubic splines for the other PAEs and MAFLD are presented in S1 Fig.

### Stratification analysis of age

For the eleven PAEs, we performed an analysis of age stratification (S3 Table). We found a statistical relationship between MiBP (RR,95%CI: Q3: 1.42, 1.04–1.93), MEOHP (RR,95%CI: Q2: 1.45, 1.05–2.00) and MAFLD in the ≥60 year-old group. In the 40–59 year-old group, higher quartiles of MECPP (RR,95%CI: Q4: 1.91, 1.13–3.26; Q3: 1.69, 1.10–2.62; *P*-trend = 0.014), MEP (RR,95%CI: Q4: 1.53, 1.06–2.21; Q3: 1.60, 1.10–2.34; Q2: 1.62, 1.10–2.38; *P*-trend = 0.079), MEHHP (RR,95%CI: Q4: 2.04, 1.22–3.41; Q3: 1.77, 1.15–2.73; *P*-trend = 0.004), MEOHP (RR,95%CI: Q4: 2.14, 1.29–3.56; Q3: 1.82, 1.18–2.79; Q2: 1.90, 1.30–2.78; *P*-trend = 0.007) and MBzP (RR,95%CI: Q4: 2.18, 1.48–3.21; Q2: 1.47, 1.06–2.04; *P*-trend = 0.001) were positively associated with MAFLD. Additionally, MnBP (RR,95%CI: Q4: 2.02, 1.02–4.01; Q3: 2.02, 1.20–3.40; *P*-trend = 0.025) and MiBP (RR,95%CI: Q4: 1.95, 1.05–3.60; Q3: 2.07, 1.25–3.44; Q2: 1.67, 1.10–2.52; *P*-trend = 0.041) were significant positively associated with MAFLD in the 20–39 year-old group.

### Stratification analysis of sex

Stratification analysis by the sex of the eleven PAEs is shown in S4 Table. Individuals in the higher quartiles of MECPP (RR,95%CI: Q4: 1.59, 1.03–2.46; Q3: 1.57, 1.08–2.29; *P*-trend = 0.025), MnBP (RR,95%CI: Q4: 1.78, 1.06–3.00; Q3: 1.59, 1.09–2.30; *P*-trend = 0.021), MEHHP (RR,95%CI: Q4: 1.82, 1.18–2.82; Q3: 1.70, 1.14–2.54; *P*-trend = 0.003), and MiBP (RR,95%CI: Q4: 1.73, 1.17–2.54; Q3: 2.10, 1.51–2.94; *P*-trend<0.001) were positively associated with MAFLD in women, while significant associations for MBzP (RR,95%CI: Q4: 1.78, 1.25–2.55; Q2: 1.50, 1.09–2.06; *P*-trend = 0.021) were observed in males.

### Association of PAEs mixture exposure and MAFLD

**Fig 4:**
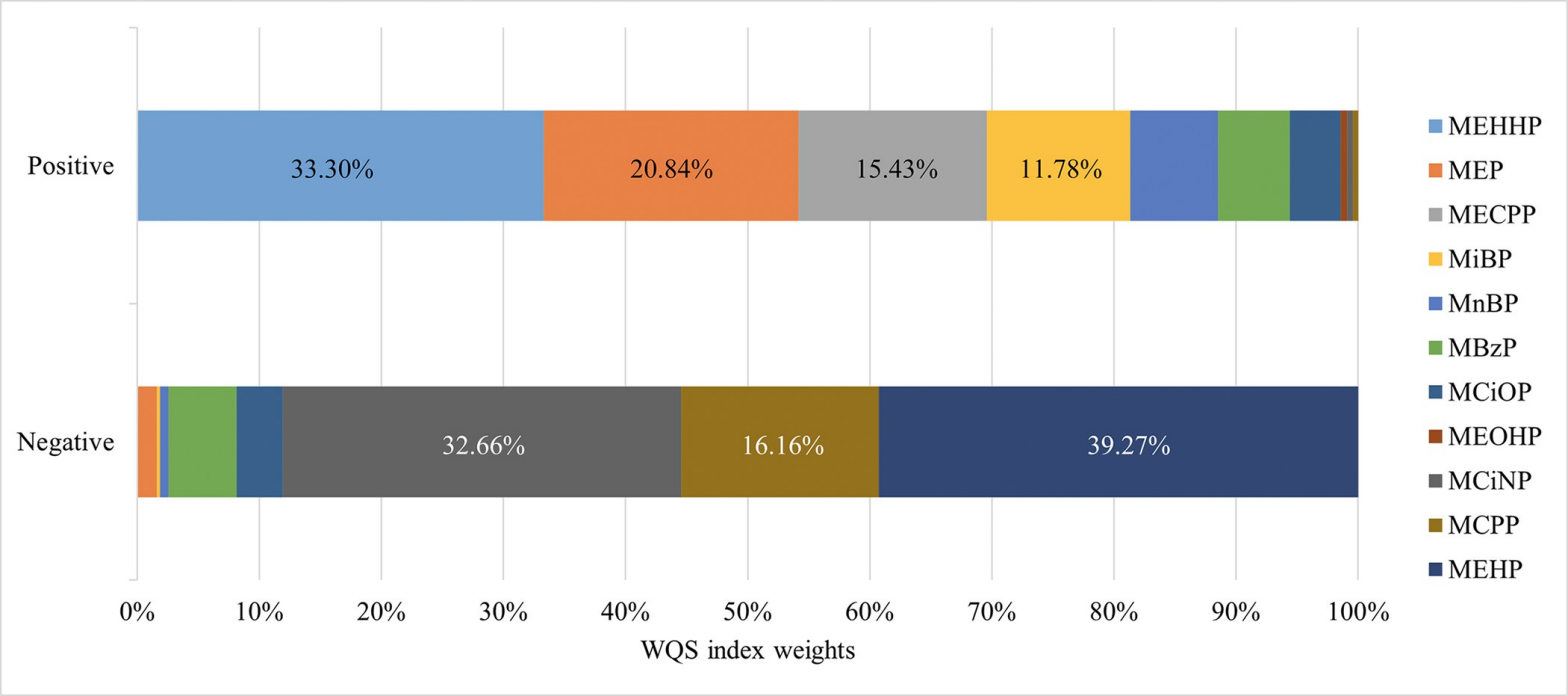
The WQS index weights of each of the eleven PAEs associated with MAFLD.

The combined effect of PAEs on MAFLD, as assessed by WQS, indicated a positive and significant association between the WQS index for the eleven PAEs and MAFLD (OR: 1.43; 95% CI: 1.20–1.70; *P* < 0.001) (Table 4). MEHHP (33.30%), MEP (20.84%), MECPP (15.43%), and MiBP (11.78%) drove the mixture effect in this model. The WQS index weights of each of the eleven PAEs associated with MAFLD are shown in Fig 4.

**Table 4:** Estimation of the mixture effect of PAEs on the risk of MAFLD using WQS.

Related Directions	OR (95%CI)	*P*-value	Component	Weights (%)
**Positive**	1.43(1.20,1.70)	<0.001	MEHHP	33.30
			MEP	20.84
			MECPP	15.43
			MiBP	11.78
			MnBP	7.17
			MBzP	5.89
			MCiOP	4.14
			MEOHP	0.55
			MCiNP	0.49
			MCPP	0.41
			MEHP	0.00
**Negative**	1.06(0.92,1.24)	0.405	MEHP	39.27
			MCiNP	32.66
			MCPP	16.16
			MBzP	5.56
			MCiOP	3.79
			MEP	1.60
			MnBP	0.69
			MiBP	0.28
			MECPP	0.00
			MEOHP	0.00
			MEHHP	0.00

Weight (%) shows individual contributions to the WQS index.

WQS: Weighted Quantile Sum.

## Discussion

The study revealed a significant association between phthalate metabolites, specifically MCiNP, MECPP, MnBP, MCPP, MEP, MEHHP, MEOHP, and MBzP, and an elevated risk of MAFLD in American adults using NHANES data from 2007 to 2018. The RCS results indicated dose-response relationships between MnBP, MEP, MEHHP, MiBP, MEOHP, MBzP, and MAFLD. Notably, mixed phthalate exposure significantly elevated the risk of MAFLD, with MEHHP being the primary contributor.

The liver serves as a crucial defense mechanism against external harmful substances. MAFLD encompasses patients with obesity, metabolic syndrome, T2DM, or any indications of metabolic dysregulation. In contrast, the traditional definition of NAFLD primarily focuses on excluding other causes of liver steatosis [24]. Previous studies have explored the connection between phthalates and NAFLD [23,32,33]. However, few scholars have studied the relationship between phthalate exposure and the prevalence of MAFLD.

A previous cross-sectional study involving 4,206 Americans revealed an association between higher MEHHP quartiles and higher odds ratios (ORs) for NAFLD, as defined by US FLI [33]. Fewer epidemiologic studies have investigated the relationship between phthalates and NAFLD as diagnosed by vibration-controlled transient elastography. A recent study involving 1450 American adults demonstrated significantly higher odds ratios (ORs) for NAFLD in the higher quartiles of MECPP and MEHHP [23]. This is consistent with the findings of our study on MAFLD, despite the different diagnostic criteria between MAFLD and NAFLD. A recent study utilizing NHANES 2017–2018 data highlighted a strong link between phthalate exposure and MAFLD, aligning with our research outcomes [34].

MEP is a key metabolite of DEP in vivo and serves as a biomarker for measuring DEP exposure. While acute human exposure to DEP has been reported as low in toxicity, persistent daily exposure persists due to the extensive use of DEP in personal care items as denaturants and fixatives [35,36]. An animal experiment report demonstrated that prolonged exposure to DEP, even at lower levels, could induce hyperglycemia and hyperinsulinemia, both indicative of insulin resistance, in mice [13]. Moreover, a separate animal study reported significant lipid peroxidation in the livers of rats treated with DEP [17]. Simultaneously, a study has indicated that adult zebrafish exposed to DEP in water can result in severe weight loss, hypolipidemia, hypoglycemia, and increased hepatic inflammation [14]. The results of multiple studies indicate that DEP-induced oxidative stress is associated with dysfunction of high-density lipoprotein (HDL), hepatic inflammation, and growth retardation [37,38]. In 2018, a study suggested that exposure to phthalates could impair liver function in males and indicated that phthalates might decrease the catalytic efficiency of cytochrome P450. Besides, the study also suggested that MEP level was associated with the increase in ALT, AST, GGT, hypertriglyceridemia, and the decrease in HDL-c [39]. Therefore, exposure to phthalates may promote the development of MAFLD.

MEHP, a primary metabolite of DEHP in the human body, did not exhibit a significant association with MAFLD in this study. However, secondary DEHP metabolites (MECPP, MEOHP, and MEHHP) displayed a notable link with MAFLD. DEHP, as the most widely used phthalate ester, has been listed as a priority pollutant by the Environmental Protection Agency (EPA) [40]. Lipases rapidly metabolize DEHP into MEHP, which is predominantly absorbed by tissues and is linked to metabolic diseases, cancer, and cardiovascular issues [41,42]. Several laboratory studies suggested that exposure to DEHP and its metabolites can influence liver lipid metabolism, leading to hepatic steatosis. A study indicated that in chronic DEHP-exposed hepatic stellate cells and mice, significant steatosis and elevated liver cholesterol levels were observed [43]. DEHP exposure may affect cholesterol metabolism. A study found that DEHP-exposed, apolipoprotein-E-deficient mice may have hypercholesterolemia and fatty livers [44]. Ioannou and Lee respectively reported that chronic low-dose DEHP exposure decreases ABCG1, the cholesterol excretion receptor. Besides, DEHP exposure increases the expression of HMGCR and SREBP2, one of the major reasons for cholesterol accumulation is the increased synthesis of endogenous cholesterol [43,45]. The results of these animal experiments and mechanistic studies indirectly support our findings. One noteworthy mechanism deserving careful consideration is the potential role of thyroid function in mediating the association between DEHP and MAFLD. DEHP may lead to thyroid dysfunction, as it possesses thyroid receptor (TR) antagonistic activity [46]. Hence, DEHP may disrupt thyroid function and subsequently induce MAFLD. However, not all studies have found a connection between DEHP and thyroid hormones, further research is needed to investigate this potential mechanism [47].

In the sex-stratified analysis of this study, the metabolites of DEHP (MEHHP and MECPP) had a significant impact on the risk of MAFLD in females, while their effect on males was not significant. Animal experiments have demonstrated that female mice have a higher risk of developing T2DM, metabolic disorders, cardiovascular events, and hepatotoxicity compared to male mice [48]. In an animal study involving DEHP exposure, female mice exhibited significantly impaired glucose tolerance associated with decreased estrogen signaling in the liver [49]. Several articles have shown that elevated estrogen activity in the livers of female mice deficient in estrogen, estrogens offer protection against metabolic-related diseases within a specific physiological range, which may explain the adverse effects of PAEs’ estrogenic activity on hepatic insulin sensitivity and glucose tolerance in adult females [49–52]. However, the specific mechanisms underlying sex differences remain unclear and require further investigation.

This study has several advantages. Firstly, it utilized data from NHANES, a nationally representative United States database. Stringent quality control measures were implemented to guarantee the authenticity and generalizability of the results. Secondly, to evaluate the potential combined impacts of different PAE metabolites, the study used WQS regression as a multiple pollutant model. This method offers insightful information regarding the MAFLD caused by the PAE mixtures. However, our study is not without limitations. Firstly, the cross-sectional design limits the confirmation of causal relationships. Further research employing case-control or cohort studies is necessary. Secondly, while US FLI proves practical and ethical for extensive epidemiological studies, invasive liver biopsy remains the gold standard for diagnosing fatty liver. Lastly, Single-spot urinary phthalate measurements might not adequately capture continuous long-term exposure and intra-individual variability, despite being suggested as a useful approach in environmental epidemiology studies [53].

## Conclusions

The findings of our research demonstrated positive associations between phthalate exposure and MAFLD in American adults, both individually and in combination. However, to establish a causal relationship, further longitudinal studies and investigation of biological mechanisms are imperative.

## Supporting information

S1 Fig Associations between PAE concentration and MAFLD in restricted cubic spline model for the overall population. The solid line and dashed lines represent the estimated ORs and the 95%CI.
(TIF)

S1 Table The classifications of categorical covariates. PIR: poverty income ratio; MET: metabolic equivalents.
(DOCX)

S2 Table Physical examination and biochemical measures in the general characteristics of the participants. WC: waist circumference; WHtR: waist-height ratio; HOMA-IR, homeostasis model assessment for insulin resistance; HDL, high-density lipoprotein; TG, triacylglycerol.
(DOCX)

S3 Table Effects estimates and 95% confidence intervals (95% CI) between MAFLD and PAEs in different age. Adjusted by age, sex, race/ ethnicity, educational level, smoking status, alcohol consumption, physical activity, PIR, creatinine, and cycle.
(DOCX)

S4 Table Effects estimates and 95% confidence intervals (95% CI) between MAFLD and PAEs in different sexes. Adjusted by age, sex, race/ ethnicity, educational level, smoking status, alcohol consumption, physical activity, PIR, creatinine, and cycle.
(DOCX)

S1 File Data file for this study. (XLSX)

## References

[1] Eslam M, Newsome PN, Sarin SK, Anstee QM, Targher G, Romero-Gomez M, et al. A new definition for metabolic dysfunction-associated fatty liver disease: An international expert consensus statement. J Hepatol. 2020;73(1):202–9. doi: 10.1016/j.jhep.2020.03.039 . 32278004

[2] Younossi ZM, Rinella ME, Sanyal AJ, Harrison SA, Brunt EM, Goodman Z, et al. From NAFLD to MAFLD: Implications of a Premature Change in Terminology. Hepatology. 2021;73(3):1194–8. doi: 10.1002/hep.31420 . 32544255

[3] Chan KE, Koh TJL, Tang ASP, Quek J, Yong JN, Tay P, et al. Global Prevalence and Clinical Characteristics of Metabolic-associated Fatty Liver Disease: A Meta-Analysis and Systematic Review of 10 739 607 Individuals. J Clin Endocrinol Metab. 2022;107(9):2691–700. doi: 10.1210/clinem/dgac321 . 35587339

[4] Liu J, Ayada I, Zhang X, Wang L, Li Y, Wen T, et al. Estimating Global Prevalence of Metabolic Dysfunction-Associated Fatty Liver Disease in Overweight or Obese Adults. Clin Gastroenterol Hepatol. 2022;20(3):e573–e82. doi: 10.1016/j.cgh.2021.02.030 . 33618024

[5] Sree CG, Buddolla V, Lakshmi BA, Kim YJ. Phthalate toxicity mechanisms: An update. Comp Biochem Physiol C Toxicol Pharmacol. 2023;263:109498. doi: 10.1016/j.cbpc.2022.109498 . 36374650

[6] Begum TF, Carpenter D. Health effects associated with phthalate activity on nuclear receptors. Rev Environ Health. 2022;37(4):567–83. doi: 10.1515/reveh-2020-0162 . 34592072

[7] Mondal T, Mondal S, Ghosh SK, Pal P, Soren T, Pandey S, et al. Phthalates—A family of plasticizers, their health risks, phytotoxic effects, and microbial bioaugmentation approaches. Environ Res. 2022;214(Pt 3):114059. doi: 10.1016/j.envres.2022.114059 . 35961545

[8] Benjamin S, Pradeep S, Josh MS, Kumar S, Masai E. A monograph on the remediation of hazardous phthalates. J Hazard Mater. 2015;298:58–72. doi: 10.1016/j.jhazmat.2015.05.004 . 26004054

[9] Wormuth M, Scheringer M, Vollenweider M, Hungerbuhler K. What are the sources of exposure to eight frequently used phthalic acid esters in Europeans? Risk Anal. 2006;26(3):803–24. doi: 10.1111/j.1539-6924.2006.00770.x . 16834635

[10] Abdel daiem MM, Rivera-Utrilla J, Ocampo-Perez R, Mendez-Diaz JD, Sanchez-Polo M. Environmental impact of phthalic acid esters and their removal from water and sediments by different technologies—a review. J Environ Manage. 2012;109:164–78. doi: 10.1016/j.jenvman.2012.05.014 . 22796723

[11] Gao DW, Wen ZD. Phthalate esters in the environment: A critical review of their occurrence, biodegradation, and removal during wastewater treatment processes. Sci Total Environ. 2016;541:986–1001. doi: 10.1016/j.scitotenv.2015.09.148 . 26473701

[12] Singh S, Li SS. Phthalates: toxicogenomics and inferred human diseases. Genomics. 2011;97(3):148–57. doi: 10.1016/j.ygeno.2010.11.008 . 21156202

[13] Mondal S, Mukherjee S. Long-term dietary administration of diethyl phthalate triggers loss of insulin sensitivity in two key insulin target tissues of mice. Hum Exp Toxicol. 2020;39(7):984–93. doi: 10.1177/0960327120909526 . 32129097

[14] Kim SM, Yoo JA, Baek JM, Cho KH. Diethyl phthalate exposure is associated with embryonic toxicity, fatty liver changes, and hypolipidemia via impairment of lipoprotein functions. Toxicol In Vitro. 2015;30(1 Pt B):383–93. doi: 10.1016/j.tiv.2015.09.026 . 26423653

[15] Cho HC. Prevalence and Factors Associated with Nonalcoholic Fatty Liver Disease in a Nonobese Korean Population. Gut Liver. 2016;10(1):117–25. doi: 10.5009/gnl14444 . 26260755 PMC4694743

[16] Feldman A, Eder SK, Felder TK, Kedenko L, Paulweber B, Stadlmayr A, et al. Clinical and Metabolic Characterization of Lean Caucasian Subjects With Non-alcoholic Fatty Liver. Am J Gastroenterol. 2017;112(1):102–10. doi: 10.1038/ajg.2016.318 . 27527746

[17] Pereira C, Rao CV. Combined and individual administration of diethyl phthalate and polychlorinated biphenyls and its toxicity in female Wistar rats. Environ Toxicol Pharmacol. 2006;21(1):93–102. doi: 10.1016/j.etap.2005.08.001 . 21783644

[18] Yu L, Yang M, Cheng M, Fan L, Wang X, Xu T, et al. Associations between urinary phthalate metabolite concentrations and markers of liver injury in the US adult population. Environ Int. 2021;155:106608. doi: 10.1016/j.envint.2021.106608 . 33964644

[19] Gallo V, Leonardi G, Genser B, Lopez-Espinosa MJ, Frisbee SJ, Karlsson L, et al. Serum perfluorooctanoate (PFOA) and perfluorooctane sulfonate (PFOS) concentrations and liver function biomarkers in a population with elevated PFOA exposure. Environ Health Perspect. 2012;120(5):655–60. doi: 10.1289/ehp.1104436 . 22289616 PMC3346788

[20] Newsome PN, Cramb R, Davison SM, Dillon JF, Foulerton M, Godfrey EM, et al. Guidelines on the management of abnormal liver blood tests. Gut. 2018;67(1):6–19. doi: 10.1136/gutjnl-2017-314924 . 29122851 PMC5754852

[21] Ungprasert P, Crowson CS, Simonetto DA, Matteson EL. Clinical Characteristics and Outcome of Hepatic Sarcoidosis: A Population-Based Study 1976–2013. Am J Gastroenterol. 2017;112(10):1556–63. doi: 10.1038/ajg.2017.231 . 28872150 PMC5629110

[22] Milosevic N, Milanovic M, Sudji J, Bosic Zivanovic D, Stojanoski S, Vukovic B, et al. Could phthalates exposure contribute to the development of metabolic syndrome and liver disease in humans? Environ Sci Pollut Res Int. 2020;27(1):772–84. doi: 10.1007/s11356-019-06831-2 . 31808097

[23] Chen X, Tian F, Wu J, Liu L, Li Y, Yu G, et al. Associations of phthalates with NAFLD and liver fibrosis: A nationally representative cross-sectional study from NHANES 2017 to 2018. Front Nutr. 2022;9:1059675. doi: 10.3389/fnut.2022.1059675 . 36483930 PMC9723339

[24] Lim GEH, Tang A, Ng CH, Chin YH, Lim WH, Tan DJH, et al. An Observational Data Meta-analysis on the Differences in Prevalence and Risk Factors Between MAFLD vs NAFLD. Clin Gastroenterol Hepatol. 2023;21(3):619–29 e7. doi: 10.1016/j.cgh.2021.11.038 . 34871813

[25] Hatch EE, Nelson JW, Qureshi MM, Weinberg J, Moore LL, Singer M, et al. Association of urinary phthalate metabolite concentrations with body mass index and waist circumference: a cross-sectional study of NHANES data, 1999–2002. Environ Health. 2008;7:27. doi: 10.1186/1476-069X-7-27 . 18522739 PMC2440739

[26] James-Todd TM, Huang T, Seely EW, Saxena AR. The association between phthalates and metabolic syndrome: the National Health and Nutrition Examination Survey 2001–2010. Environ Health. 2016;15:52. doi: 10.1186/s12940-016-0136-x . 27079661 PMC4832560

[27] Trasande L, Sathyanarayana S, Spanier AJ, Trachtman H, Attina TM, Urbina EM. Urinary phthalates are associated with higher blood pressure in childhood. J Pediatr. 2013;163(3):747–53 e1. doi: 10.1016/j.jpeds.2013.03.072 . 23706605 PMC4074773

[28] Ruhl CE, Everhart JE. Fatty liver indices in the multiethnic United States National Health and Nutrition Examination Survey. Aliment Pharmacol Ther. 2015;41(1):65–76. doi: 10.1111/apt.13012 . 25376360

[29] Committee ADAPP. 2. Classification and Diagnosis of Diabetes: Standards of Medical Care in Diabetes-2022. Diabetes Care. 2022;45(Suppl 1):S17–S38. doi: 10.2337/dc22-S002 . 34964875

[30] Keil AP, Buckley JP, O’Brien KM, Ferguson KK, Zhao S, White AJ. A Quantile-Based g-Computation Approach to Addressing the Effects of Exposure Mixtures. Environ Health Perspect. 2020;128(4):47004. doi: 10.1289/EHP5838 . 32255670 PMC7228100

[31] Carrico C, Gennings C, Wheeler DC, Factor-Litvak P. Characterization of Weighted Quantile Sum Regression for Highly Correlated Data in a Risk Analysis Setting. J Agric Biol Environ Stat. 2015;20(1):100–20. doi: 10.1007/s13253-014-0180-3 . 30505142 PMC6261506

[32] Li W, Xiao H, Wu H, Pan C, Deng K, Xu X, et al. Analysis of environmental chemical mixtures and nonalcoholic fatty liver disease: NHANES 1999–2014. Environ Pollut. 2022;311:119915. doi: 10.1016/j.envpol.2022.119915 . 35970346

[33] Cai S, Fan J, Ye J, Rao X, Li Y. Phthalates exposure is associated with non-alcoholic fatty liver disease among US adults. Ecotoxicol Environ Saf. 2021;224:112665. doi: 10.1016/j.ecoenv.2021.112665 . 34438269

[34] Lei R, Xue B, Tian X, Liu C, Li Y, Zheng J, et al. The association between endocrine disrupting chemicals and MAFLD: Evidence from NHANES survey. Ecotoxicol Environ Saf. 2023;256:114836. doi: 10.1016/j.ecoenv.2023.114836 . 37001192

[35] Janjua NR, Mortensen GK, Andersson AM, Kongshoj B, Skakkebaek NE, Wulf HC. Systemic uptake of diethyl phthalate, dibutyl phthalate, and butyl paraben following whole-body topical application and reproductive and thyroid hormone levels in humans. Environ Sci Technol. 2007;41(15):5564–70. doi: 10.1021/es0628755 . 17822133

[36] Hauser R, Calafat AM. PHTHALATES AND HUMAN HEALTH. Occup Environ Med. 2005;62(11):806–18. doi: 10.1136/oem.2004.017590 . 16234408 PMC1740925

[37] Pereira C, Mapuskar K, Rao CV. Chronic toxicity of diethyl phthalate in male Wistar rats—a dose-response study. Regul Toxicol Pharmacol. 2006;45(2):169–77. doi: 10.1016/j.yrtph.2006.04.006 . 16750591

[38] Ghorpade N, Mehta V, Khare M, Sinkar P, Krishnan S, Rao CV. Toxicity study of diethyl phthalate on freshwater fish Cirrhina mrigala. Ecotoxicol Environ Saf. 2002;53(2):255–8. doi: 10.1006/eesa.2002.2212 . 12568461

[39] Milosevic N, Milic N, Zivanovic Bosic D, Bajkin I, Percic I, Abenavoli L, et al. Potential influence of the phthalates on normal liver function and cardiometabolic risk in males. Environ Monit Assess. 2017;190(1):17. doi: 10.1007/s10661-017-6398-0 . 29234897

[40] Wang J, Chen G, Christie P, Zhang M, Luo Y, Teng Y. Occurrence and risk assessment of phthalate esters (PAEs) in vegetables and soils of suburban plastic film greenhouses. Sci Total Environ. 2015;523:129–37. doi: 10.1016/j.scitotenv.2015.02.101 . 25863503

[41] Hanioka N, Isobe T, Kinashi Y, Tanaka-Kagawa T, Jinno H. Hepatic and intestinal glucuronidation of mono(2-ethylhexyl) phthalate, an active metabolite of di(2-ethylhexyl) phthalate, in humans, dogs, rats, and mice: an in vitro analysis using microsomal fractions. Arch Toxicol. 2016;90(7):1651–7. doi: 10.1007/s00204-015-1619-1 . 26514348

[42] Koch HM, Bolt HM, Angerer J. Di(2-ethylhexyl)phthalate (DEHP) metabolites in human urine and serum after a single oral dose of deuterium-labelled DEHP. Arch Toxicol. 2004;78(3):123–30. doi: 10.1007/s00204-003-0522-3 . 14576974

[43] Lee CY, Suk FM, Twu YC, Liao YJ. Long-Term Exposure to Low-Dose Di-(2-ethylhexyl) Phthalate Impairs Cholesterol Metabolism in Hepatic Stellate Cells and Exacerbates Liver Librosis. Int J Environ Res Public Health. 2020;17(11). doi: 10.3390/ijerph17113802 . 32471116 PMC7312183

[44] Zhao JF, Hsiao SH, Hsu MH, Pao KC, Kou YR, Shyue SK, et al. Di-(2-ethylhexyl) phthalate accelerates atherosclerosis in apolipoprotein E-deficient mice. Arch Toxicol. 2016;90(1):181–90. doi: 10.1007/s00204-014-1377-5 . 25270622

[45] Ioannou GN. The Role of Cholesterol in the Pathogenesis of NASH. Trends Endocrinol Metab. 2016;27(2):84–95. doi: 10.1016/j.tem.2015.11.008 . 26703097

[46] Shen O, Du G, Sun H, Wu W, Jiang Y, Song L, et al. Comparison of in vitro hormone activities of selected phthalates using reporter gene assays. Toxicol Lett. 2009;191(1):9–14. doi: 10.1016/j.toxlet.2009.07.019 . 19643168

[47] Tsai HJ, Wu CF, Tsai YC, Huang PC, Chen ML, Wang SL, et al. Intake of Phthalate-tainted Foods and Serum Thyroid Hormones in Taiwanese Children and Adolescents. Sci Rep. 2016;6:30589. doi: 10.1038/srep30589 . 27470018 PMC4965773

[48] Ding Y, Xu T, Mao G, Chen Y, Qiu X, Yang L, et al. Di-(2-ethylhexyl) phthalate-induced hepatotoxicity exacerbated type 2 diabetes mellitus (T2DM) in female pubertal T2DM mice. Food Chem Toxicol. 2021;149:112003. doi: 10.1016/j.fct.2021.112003 . 33484791

[49] Naville D, Pinteur C, Vega N, Menade Y, Vigier M, Le Bourdais A, et al. Low-dose food contaminants trigger sex-specific, hepatic metabolic changes in the progeny of obese mice. Faseb j. 2013;27(9):3860–70. doi: 10.1096/fj.13-231670 . 23756648

[50] Gao J, He J, Shi X, Stefanovic-Racic M, Xu M, O’Doherty RM, et al. Sex-specific effect of estrogen sulfotransferase on mouse models of type 2 diabetes. Diabetes. 2012;61(6):1543–51. doi: 10.2337/db11-1152 . 22438574 PMC3357292

[51] Naville D, Labaronne E, Vega N, Pinteur C, Canet-Soulas E, Vidal H, et al. Metabolic outcome of female mice exposed to a mixture of low-dose pollutants in a diet-induced obesity model. PLoS One. 2015;10(4):e0124015. doi: 10.1371/journal.pone.0124015 . 25909471 PMC4409066

[52] Julien B, Pinteur C, Vega N, Labaronne E, Vidal H, Naville D, et al. Evidence for estrogeno-mimetic effects of a mixture of low-dose pollutants in a model of ovariectomized mice. Environ Toxicol Pharmacol. 2018;57:34–40. doi: 10.1016/j.etap.2017.11.008 . 29175711

[53] Johns LE, Cooper GS, Galizia A, Meeker JD. Exposure assessment issues in epidemiology studies of phthalates. Environ Int. 2015;85:27–39. doi: 10.1016/j.envint.2015.08.005 . 26313703 PMC4648682

