# Supplementary figures and images for: Relationship between phthalates exposures and metabolic dysfunction-associated fatty liver disease in United States adults
Source: PLoS One. 2024 Apr 19;19(4):e0301097. doi: 10.1371/journal.pone.0301097 (PMC11029636; doi:10.1371/journal.pone.0301097)

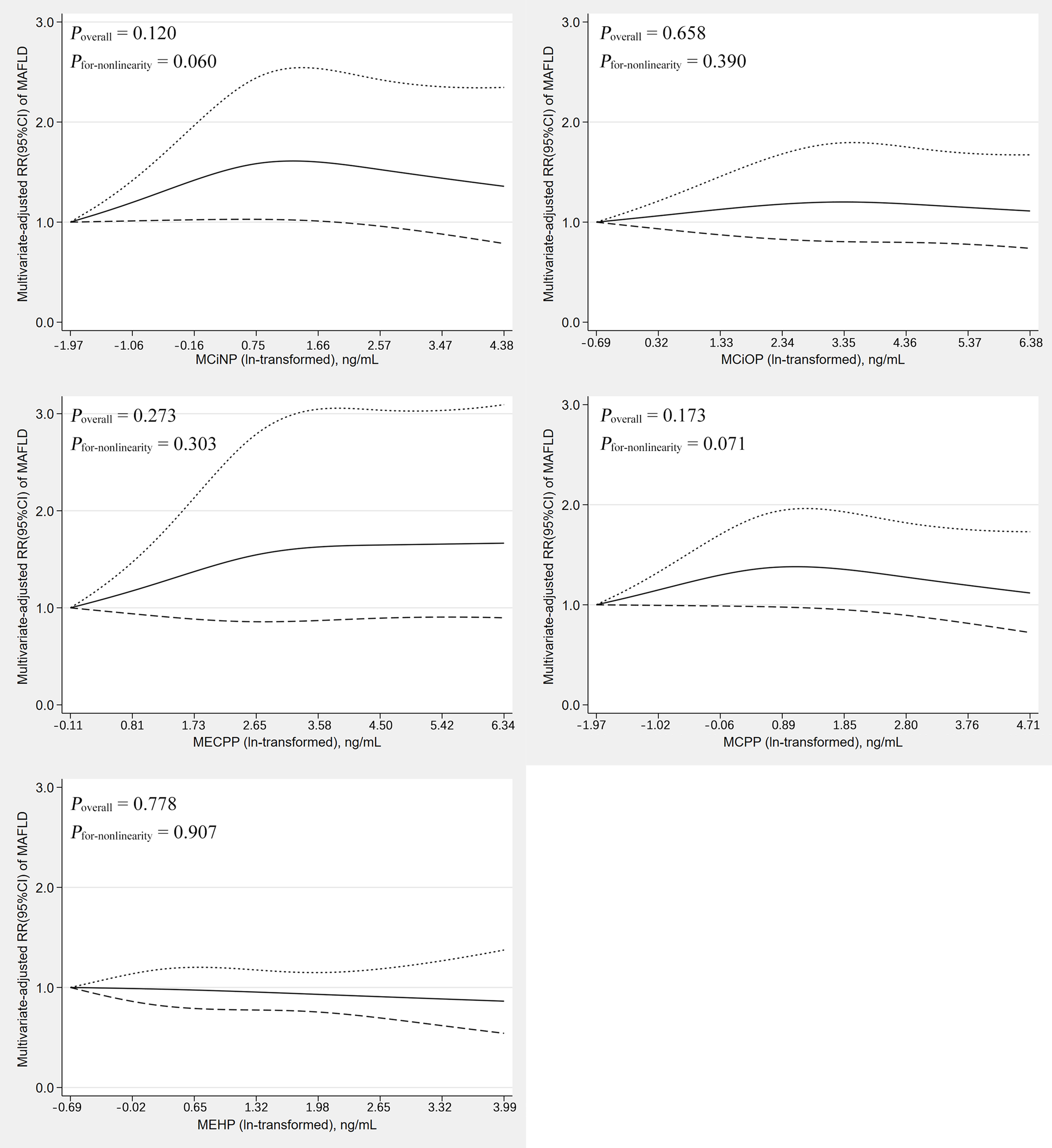

Supplement: S1 Fig — The solid line and dashed lines represent the estimated ORs and the 95%CI. (TIF) [file pone.0301097.s001.tif]
