# Supplementary material for: Relationship between phthalates exposures and metabolic dysfunction-associated fatty liver disease in United States adults
Source: PLoS One. 2024 Apr 19;19(4):e0301097. doi: 10.1371/journal.pone.0301097 (PMC11029636; doi:10.1371/journal.pone.0301097)
Supplement: S1 Table — PIR: poverty income ratio; MET: metabolic equivalents. (DOCX) [file pone.0301097.s002.docx]

**S1 table.** **The classifications of categorical covariates.**

| **Covariates** | **Classifications** | **Definitions** |
| --- | --- | --- |
| **Education levels** | <high school |  |
|  | high school or equivalent |  |
|  | >high school |  |
| **Household income levels** | low | PIR<1.30 |
|  | middle | middle (1.30≤PIR≤3.50) |
|  | high | PIR>3.50 |
| **Smoke status** | never smokers | those who never smoked or smoked less than 100 cigarettes |
|  | former smokers | those who had smoked at least 100 cigarettes in their lifetime but had quit smoking for more than six months |
|  | current smokers | those who had smoked at least 100 cigarettes, currently smoking or had quit smoking less than six months |
| **Alcohol consumption** | never drinkers |  |
|  | moderate drinkers | 1-2 drinks/day for males, 1 drink/day for females |
|  | heavy drinkers | >2 drinks/day for males, >1drink/day for females |
| **Physical activity quantities** | sedentary | no physical activity |
|  | insufficient | some physical activity but less than 500 MET-minutes per week |
|  | moderate | ≥500 and <1000 MET-minutes per week |

PIR: poverty income ratio; MET: metabolic equivalents.
