# Supplementary material for: Relationship between phthalates exposures and metabolic dysfunction-associated fatty liver disease in United States adults
Source: PLoS One. 2024 Apr 19;19(4):e0301097. doi: 10.1371/journal.pone.0301097 (PMC11029636; doi:10.1371/journal.pone.0301097)
Supplement: S2 Table — WC: waist circumference; WHtR: waist-height ratio; HOMA-IR, homeostasis model assessment for insulin resistance; HDL, high-density lipoprotein; TG, triacylglycerol. (DOCX) [file pone.0301097.s003.docx]

**S2 Table.** **Physical examination and biochemical measures in the general characteristics of the participants.**

| **Variables** | | **Total** | | **non-MAFLD** | | **MAFLD** | | ***P* value** | |
| --- | --- | --- | --- | --- | --- | --- | --- | --- | --- |
| **WC (cm) ^a^** | | 97.60(21.80) | | 91.60(17.30) | | 110.80(17.90) | | ＜0.001 | |
| **WHtR ^a^** | | 0.58(0.13) | | 0.55(0.10) | | 0.66(0.11) | | ＜0.001 | |
| **HOMA-IR ^a^** | | 2.50(2.92) | | 1.77(1.37) | | 5.33(4.31) | | ＜0.001 | |
| **HDL (mg/dl) ^a^** | | 52.00(20.00) | | 56.00(22.00) | | 45.00(14.00) | | ＜0.001 | |
| **TG (mg/dl) ^a^** | | 99.00(77.00) | | 86.00(57.00) | | 137.00(92.00) | | ＜0.001 | |

WC: waist circumference; WHtR: waist-height ratio; HOMA-IR, homeostasis model assessment for insulin resistance; HDL, high-density lipoprotein; TG, triacylglycerol.
