# Supplementary material for: Relationship between phthalates exposures and metabolic dysfunction-associated fatty liver disease in United States adults
Source: PLoS One. 2024 Apr 19;19(4):e0301097. doi: 10.1371/journal.pone.0301097 (PMC11029636; doi:10.1371/journal.pone.0301097)
Supplement: S3 Table — Adjusted by age, sex, race/ ethnicity, educational level, smoking status, alcohol consumption, physical activity, PIR, creatinine, and cycle. (DOCX) [file pone.0301097.s004.docx]

**S2 Table. Effects estimates and 95% confidence intervals (95% CI) between MAFLD and PAEs in different age.**

|  |  | **20–39** | |  | **40–59** | |  | **≥60** | |
| --- | --- | --- | --- | --- | --- | --- | --- | --- | --- |
|  |  | **RR (95% CI)** | ***P*-trend** |  | **RR (95% CI)** | ***P*-trend** |  | **RR (95% CI)** | ***P*-trend** |
| **MCiNP** | Q1 | Ref. | 0.275 |  | Ref. | 0.344 |  | Ref. | 0.985 |
|  | Q2 | 1.29(0.80,2.07) |  |  | 1.32(0.91,1.91) |  |  | 1.20(0.88,1.64) |  |
|  | Q3 | 1.62(1.00,2.60)^*^ |  |  | 1.42(1.02,1.97)^*^ |  |  | 0.97(0.67,1.41) |  |
|  | Q4 | 1.30(0.78,2.17) |  |  | 1.23(0.79,1.91) |  |  | 1.09(0.76,1.57) |  |
| **MCiOP** | Q1 | Ref. | 0.224 |  | Ref. | 0.457 |  | Ref. | 0.987 |
|  | Q2 | 1.64(1.07,2.51)^*^ |  |  | 1.32(0.94,1.84) |  |  | 1.15(0.85,1.55) |  |
|  | Q3 | 1.21(0.73,2.01) |  |  | 1.28(0.86,1.91) |  |  | 0.91(0.60,1.36) |  |
|  | Q4 | 1.70(0.97,2.98) |  |  | 1.23(0.79,1.91) |  |  | 1.08(0.75,1.57) |  |
| **MECPP** | Q1 | Ref. | 0.859 |  | Ref. | 0.014 |  | Ref. | 0.109 |
|  | Q2 | 1.24(0.79,1.95) |  |  | 1.47(0.98,2.19) |  |  | 1.25(0.92,1.69) |  |
|  | Q3 | 0.99(0.64,1.52) |  |  | 1.69(1.10,2.62)^*^ |  |  | 1.41(0.93,2.12) |  |
|  | Q4 | 1.13(0.69,1.86) |  |  | 1.91(1.13,3.26)^*^ |  |  | 1.41(0.91,2.21) |  |
| **MnBP** | Q1 | Ref. | 0.025 |  | Ref. | 0.084 |  | Ref. | 0.612 |
|  | Q2 | 1.53(0.94,2.50) |  |  | 1.39(0.96,2.00) |  |  | 0.97(0.71,1.33) |  |
|  | Q3 | 2.02(1.20,3.40)^**^ |  |  | 1.30(0.83,2.02) |  |  | 1.00(0.69,1.47) |  |
|  | Q4 | 2.02(1.02,4.01)^*^ |  |  | 1.60(0.99,2.56) |  |  | 0.82(0.52,1.30) |  |
| **MCPP** | Q1 | Ref. | 0.502 |  | Ref. | 0.131 |  | Ref. | 0.64 |
|  | Q2 | 1.71(1.09,2.69)^*^ |  |  | 1.43(0.96,2.14) |  |  | 1.23(0.93,1.61) |  |
|  | Q3 | 1.46(0.86,2.49) |  |  | 1.29(0.87,1.91) |  |  | 1.37(0.99,1.88) |  |
|  | Q4 | 1.40(0.82,2.38) |  |  | 1.56(0.93,2.62) |  |  | 1.00(0.72,1.40) |  |
| **MEP** | Q1 | Ref. | 0.564 |  | Ref. | 0.079 |  | Ref. | 0.253 |
|  | Q2 | 1.32(0.84,2.07) |  |  | 1.62(1.10,2.38)^*^ |  |  | 1.14(0.80,1.64) |  |
|  | Q3 | 1.25(0.78,2.02) |  |  | 1.60(1.10,2.34)^*^ |  |  | 1.08(0.71,1.63) |  |
|  | Q4 | 1.17(0.75,1.85) |  |  | 1.53(1.06,2.21)^*^ |  |  | 1.33(0.88,2.01) |  |
| **MEHHP** | Q1 | Ref. | 0.826 |  | Ref. | 0.004 |  | Ref. | 0.163 |
|  | Q2 | 1.22(0.73,2.03) |  |  | 1.36(0.92,2.02) |  |  | 1.33(0.96,1.85) |  |
|  | Q3 | 1.13(0.70,1.82) |  |  | 1.77(1.15,2.73)^*^ |  |  | 1.36(0.94,1.95) |  |
|  | Q4 | 1.09(0.64,1.86) |  |  | 2.04(1.22,3.41)^**^ |  |  | 1.37(0.86,2.17) |  |
| **MEHP** | Q1 | Ref. | 0.014 |  | Ref. | 0.484 |  | Ref. | 0.589 |
|  | Q2 | 0.90(0.61,1.34) |  |  | 0.90(0.62,1.30) |  |  | 1.14(0.80,1.64) |  |
|  | Q3 | 0.84(0.55,1.28) |  |  | 1.08(0.77,1.51) |  |  | 0.97(0.65,1.44) |  |
|  | Q4 | 0.55(0.35,0.85)^**^ |  |  | 1.09(0.75,1.58) |  |  | 1.23(0.80,1.88) |  |
| **MiBP** | Q1 | Ref. | 0.041 |  | Ref. | 0.224 |  | Ref. | 0.373 |
|  | Q2 | 1.67(1.10,2.52)^*^ |  |  | 0.89(0.62,1.27) |  |  | 1.18(0.90,1.54) |  |
|  | Q3 | 2.07(1.25,3.44)^**^ |  |  | 1.35(0.96,1.91) |  |  | 1.42(1.04,1.93)^*^ |  |
|  | Q4 | 1.95(1.05,3.60)^*^ |  |  | 1.12(0.76,1.65) |  |  | 1.01(0.64,1.59) |  |
| **MEOHP** | Q1 | Ref. | 0.823 |  | Ref. | 0.007 |  | Ref. | 0.478 |
|  | Q2 | 1.18(0.73,1.91) |  |  | 1.90(1.30,2.78)^**^ |  |  | 1.45(1.05,2.00)^*^ |  |
|  | Q3 | 1.07(0.70,1.64) |  |  | 1.82(1.18,2.79)^**^ |  |  | 1.17(0.75,1.83) |  |
|  | Q4 | 1.10(0.65,1.86) |  |  | 2.14(1.29,3.56)^**^ |  |  | 1.23(0.75,2.02) |  |
| **MBzP** | Q1 | Ref. | 0.014 |  | Ref. | 0.001 |  | Ref. | 0.5 |
|  | Q2 | 1.37(0.83,2.26) |  |  | 1.47(1.06,2.04)^*^ |  |  | 1.03(0.78,1.36) |  |
|  | Q3 | 1.39(0.80,2.43) |  |  | 1.29(0.92,1.82) |  |  | 0.96(0.70,1.31) |  |
|  | Q4 | 1.91(1.13,3.22)^*^ |  |  | 2.18(1.48,3.21)^**^ |  |  | 0.86(0.54,1.36) |  |

Adjusted by age, sex, race/ ethnicity, educational level, smoking status, alcohol consumption, physical activity, PIR, creatinine and cycle.
