# Supplementary material for: Relationship between phthalates exposures and metabolic dysfunction-associated fatty liver disease in United States adults
Source: PLoS One. 2024 Apr 19;19(4):e0301097. doi: 10.1371/journal.pone.0301097 (PMC11029636; doi:10.1371/journal.pone.0301097)
Supplement: S4 Table — Adjusted by age, sex, race/ ethnicity, educational level, smoking status, alcohol consumption, physical activity, PIR, creatinine, and cycle. (DOCX) [file pone.0301097.s005.docx]

**S3 Table. Effects estimates and 95% confidence intervals (95% CI) between MAFLD and PAEs in different sexes**

|  |  | **Male** |  |  | **Female** |  |
| --- | --- | --- | --- | --- | --- | --- |
|  |  | **RR (95% CI)** | ***P*-trend** |  | **RR (95% CI)** | ***P*-trend** |
| **MCiNP** | Q1 | Ref. | 0.381 |  | Ref. | 0.397 |
|  | Q2 | 1.32(0.93,1.87) |  |  | 1.18(0.83,1.68) |  |
|  | Q3 | 1.27(0.95,1.70) |  |  | 1.31(0.88,1.95) |  |
|  | Q4 | 1.23(0.88,1.72) |  |  | 1.16(0.77,1.73) |  |
| **MCiOP** | Q1 | Ref. | 0.165 |  | Ref. | 0.858 |
|  | Q2 | 1.39(1.00,1.95) |  |  | 1.21(0.90,1.62) |  |
|  | Q3 | 1.18(0.81,1.71) |  |  | 1.00(0.70,1.43) |  |
|  | Q4 | 1.40(0.97,2.02) |  |  | 1.11(0.78,1.58) |  |
| **MECPP** | Q1 | Ref. | 0.093 |  | Ref. | 0.025 |
|  | Q2 | 1.33(0.94,1.86) |  |  | 1.33(0.95,1.86) |  |
|  | Q3 | 1.25(0.90,1.74) |  |  | 1.57(1.08,2.29)^*^ |  |
|  | Q4 | 1.41(0.98,2.01) |  |  | 1.59(1.03,2.46)^*^ |  |
| **MnBP** | Q1 | Ref. | 0.367 |  | Ref. | 0.021 |
|  | Q2 | 1.23(0.96,1.58) |  |  | 1.41(0.98,2.04) |  |
|  | Q3 | 1.20(0.87,1.65) |  |  | 1.59(1.09,2.30)^*^ |  |
|  | Q4 | 1.23(0.82,1.84) |  |  | 1.78(1.06,3.00)^*^ |  |
| **MCPP** | Q1 | Ref. | 0.064 |  | Ref. |  |
|  | Q2 | 1.39(1.04,1.85)^*^ |  |  | 1.39(0.99,1.95) |  |
|  | Q3 | 1.30(0.97,1.74) |  |  | 1.40(1.03,1.89)^*^ |  |
|  | Q4 | 1.43(1.04,1.96)^*^ |  |  | 1.13(0.73,1.75) |  |
| **MEP** | Q1 | Ref. | 0.460 |  | Ref. | 0.037 |
|  | Q2 | 1.37(1.03,1.82)^*^ |  |  | 1.30(0.97,1.75) |  |
|  | Q3 | 1.03(0.77,1.39) |  |  | 1.69(1.14,2.50)^**^ |  |
|  | Q4 | 1.25(0.93,1.69) |  |  | 1.52(1.01,2.28)^*^ |  |
| **MEHHP** | Q1 | Ref. | 0.153 |  | Ref. | 0.003 |
|  | Q2 | 1.30(0.98,1.73) |  |  | 1.30(0.91,1.85) |  |
|  | Q3 | 1.27(0.95,1.70) |  |  | 1.70(1.14,2.54)^*^ |  |
|  | Q4 | 1.31(0.92,1.85) |  |  | 1.82(1.18,2.82)^**^ |  |
| **MEHP** | Q1 | Ref. | 0.935 |  | Ref. | 0.400 |
|  | Q2 | 0.93(0.72,1.19) |  |  | 1.05(0.75,1.48) |  |
|  | Q3 | 1.11(0.89,1.40) |  |  | 0.82(0.53,1.26) |  |
|  | Q4 | 0.95(0.72,1.25) |  |  | 0.92(0.60,1.40) |  |
| **MiBP** | Q1 | Ref. | 0.765 |  | Ref. | ＜0.001 |
|  | Q2 | 0.95(0.73,1.23) |  |  | 1.35(0.99,1.85) |  |
|  | Q3 | 1.09(0.80,1.47) |  |  | 2.10(1.51,2.94)^**^ |  |
|  | Q4 | 1.01(0.70,1.44) |  |  | 1.73(1.17,2.54)^**^ |  |
| **MEOHP** | Q1 | Ref. | 0.083 |  | Ref. | 0.050 |
|  | Q2 | 1.66(1.23,2.24)^**^ |  |  | 1.35(0.95,1.92) |  |
|  | Q3 | 1.33(0.98,1.80) |  |  | 1.46(1.02,2.11)^*^ |  |
|  | Q4 | 1.52(1.05,2.19)^*^ |  |  | 1.48(0.95,2.31) |  |
| **MBzP** | Q1 | Ref. | 0.021 |  | Ref. | 0.092 |
|  | Q2 | 1.50(1.09,2.06)^*^ |  |  | 1.04(0.76,1.42) |  |
|  | Q3 | 1.25(0.85,1.84) |  |  | 1.13(0.79,1.62) |  |
|  | Q4 | 1.78(1.25,2.55)^**^ |  |  | 1.40(0.95,2.05) |  |

Adjusted by age, sex, race/ ethnicity, educational level, smoking status, alcohol consumption, physical activity, PIR, creatinine and cycle.
